# Mental construction of object symbols from meaningless elements by Japanese macaques (*Macaca fuscata*)

**DOI:** 10.1038/s41598-022-07563-z

**Published:** 2022-03-04

**Authors:** Nanxi Liu, Atsuhiko Iijima, Yutaka Iwata, Kento Ohashi, Nobuyoshi Fujisawa, Toshikuni Sasaoka, Isao Hasegawa

**Affiliations:** 1 Department of Physiology, Niigata University School of Medicine, 1-757 Asahimachi St, Chuo-ku, Niigata, 951-8510 Japan; 2 Graduate School of Science and Technology, Niigata University, Niigata, Japan; 3 School of Health Sciences, Niigata University, Niigata, Japan; 4 Brain Research Institute, Niigata University, Niigata, Japan; 5 Neurophysiology & Biomedical Engineering Lab, Interdisciplinary Program of Biomedical Engineering, Assistive Technology and Art and Sports Sciences, Faculty of Engineering, Niigata University, 8050 2-no-chou, Ikarashi, Nishi-ku, Niigata, 950-2181 Japan

**Keywords:** Cognitive neuroscience, Animal behaviour

## Abstract

When writing an object’s name, humans mentally construct its spelling. This capacity critically depends on use of the dual-structured linguistic system, in which meaningful words are represented by combinations of meaningless letters. Here we search for the evolutionary origin of this capacity in primates by designing dual-structured bigram symbol systems where different combinations of meaningless elements represent different objects. Initially, we trained Japanese macaques (*Macaca fuscata*) in an object-bigram symbolization task and in a visually-guided bigram construction task. Subsequently, we conducted a probe test using a symbolic bigram construction task. From the initial trial of the probe test, the Japanese macaques could sequentially choose the two elements of a bigram that was not actually seen but signified by a visually presented object. Moreover, the animals’ spontaneous choice order bias, developed through the visually-guided bigram construction learning, was immediately generalized to the symbolic bigram construction test. Learning of dual-structured symbols by the macaques possibly indicates pre-linguistic adaptations for the ability of mentally constructing symbols in the common ancestors of humans and Old World monkeys.

## Introduction

A written message can be divided into morphemes or meaningful words (*e.g.*, “CAT”), which can be further divided into graphemes or meaningless letters (*e.g.*, “C”, “A” and “T”)^1,2^. Such a hierarchical dual structure, or duality of patterning, is one of the cardinal design features of human language which enables creation of myriads of meaningful words by combining a limited number of letters^1,3,4^. It may sound plausible that school children learn to spell such dual-structured words exclusively depending on phoneme-to-grapheme conversion following maturation of vocal/auditory language. However, recent psychological theories proposed that learning to spell begins earlier, facilitated not only by phonology^5,6^ but also by nonphonological graphotactic knowledge^7^. Scribbles of preschool children replicate smaller, darker, and more linear characteristics of writing compared to drawing^8,9^, indicating that they implicitly know that spelling is different from drawing. Toddlers as young as 2 years of age already begin to recognize statistical patterns regarding the sequence of written letters—some letters often appear together whereas others not—before they learn how letters represent the sounds of a language^10,11^. Although available evidence for how spelling gains semanticity is limited, preschool spellers might also have a naïve view that writing functions as a symbol standing for something outside itself^12^. Thus, spelling acquisition is not a rote memorization but an intricate developmental process to learn the orthographical rules governing the writing system in which graphotactic and semantic knowledge possibly plays important roles.

It has been claimed that linguistic abilities comprise faculty of language in a narrow sense (FLN) and faculty of language in a broader sense (FLB)^3,13^. Whereas FLN is uniquely human, FLB is considered as a heterogeneous collection of sensory, mnemonic, and/or cognitive skills that are often observed in infants with premature linguistic abilities as well as in some non-human animals^13^. In the present study, we aimed to search for the evolutionary origin of spelling dual-structured symbols in non-hominid primates. Previous studies showed that Japanese macaques (*Macaca fuscata*) have abilities to learn associations between visual stimuli^14,15^ and to mentally manipulate memory contents^16^. Therefore, we specifically asked whether Japanese macaques can acquire three fundamental features of human spelling required for mental symbol construction in the absence of training in phoneme-to-grapheme conversion.

First, prephonological spellers begin to recognize arbitrary patterns of letter sequences as units of written language, exhibiting bias to using specific letters (e.g., letters included in their own name) when spelling^17^. They also tend to use specific letterstrings that are more frequent than others in their mother language^10,11^. These findings indicate that acquisition of spelling might depend on experience-based statistical learning. Interestingly, Guinea baboons (*Papio papio*) can discriminate between spelling of English words and pseudo-words after repetitive exposures, presumably depending on statistical learning^18^. By repeated exposure to a sequence of visual stimuli in a fixed order, neurons in the inferior temporal cortex of Japanese macaques (*Macaca fuscata*) tend to similarly respond to two consecutive stimuli in the list regardless of their pictorial dissimilarity, indicating the relevance of statistical learning in shaping memory in the macaque brain^19^. Thus, Old World monkeys can learn to discriminate arbitrary sequence of visual elements by statistical learning. In the present study, we tested whether the macaques have the ability to combine two meaningless elements into a bigram, minimal composite symbol made of arbitrary element combination, in a visually-guided bigram construction (BC) task. In the BC task, each bigram cue specified the combination, but not the permutation of the two elements to be chosen. Therefore, the animals were allowed to voluntarily choose the two elements in any order they liked, similar to the stroke order bias observed in preschool spellers^20^. We defined a preference index as the probability of each element of the bigram to be chosen first by individual monkeys and used this index as a measure to quantify the consolidation level of the statistical learning.

Second, spelling has semanticity whereby writing units serve as symbols signifying specific referents (persons or objects)^2,3^. Here, the association between a symbol and a referent is arbitrary. Following this reasoning, children experienced with phoneme-to-grapheme conversion should be able to also advance their spelling skills by transferring linguistic knowledge to spelling. However, prephonological spellers might also implicitly know that a word functions as a symbol standing for concrete objects^21^. Previous studies have shown that macaques can learn arbitrary stimulus-stimulus associations^14,15^. In the present study, prior to the BC learning, we trained the macaques in an object bigram symbolization (OBS) task to inform the animals that bigrams symbolize artificial and natural objects including animals.

**Figure 1:**
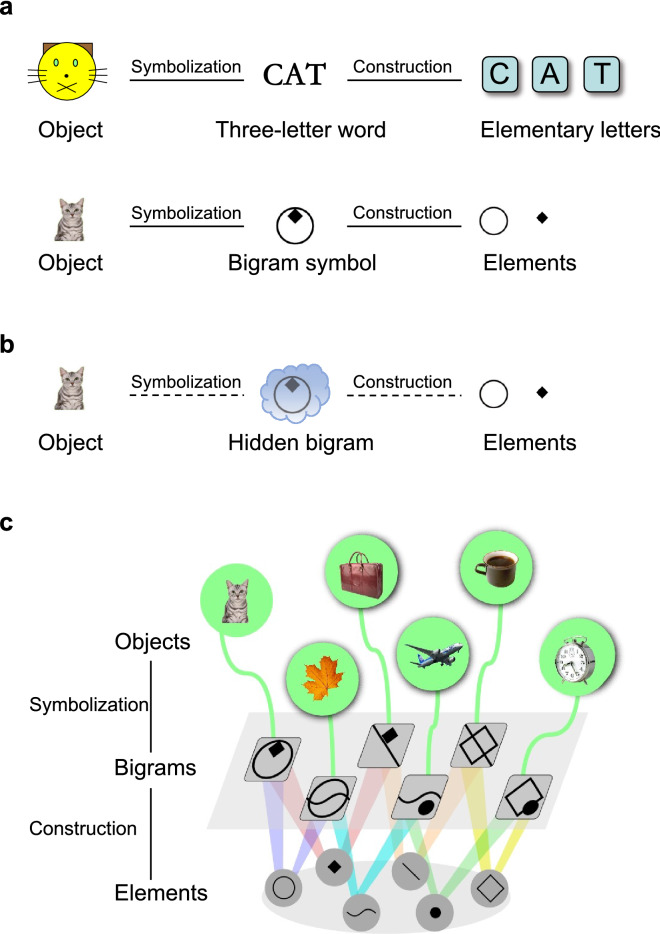
Design of a dual-structured visual symbol system. (**a**) Dual structure of written English (top) and Yerkish (bottom). An English word (e.g., “CAT”) serves a meaningful linguistic unit representing an object (a four-legged animal) and, concurrently, is a combination of meaningless letters (e.g., “C”, “A” and “T”). Similarly, a Yerkish bigram serves as a meaningful unit representing an object and is a combination of two meaningless elements. (**b**) A test paradigm examining the ability of monkeys to mentally construct dual-structured symbols. After object-bigram symbolization learning and visually guided bigram construction learning, we examined whether the monkeys could sequentially choose the elements constituting the bigram symbolically associated with an object, even when the bigram symbol itself is hidden. (**c**) Dual-structured visual symbol system. Six objects (upper) are represented by six arbitrary Yerkish bigrams (middle), which comprise different combinations of elements (lower).

Third, an experienced human adult spells an object’s name from letters even when the spelling itself is hidden, which requires an ability to mentally construct the hidden words from elements^1,3^. Previously, an ability to count the number of physically- and mentally- constructed objects has been demonstrated in parrots (*Psittacus erithacus*), dogs (*Canis lupus familiaris*) and chimpanzees (*Pan troglodytes*)^22–24^. The ability to mentally manipulate/rotate spatial working memory has also been reported in the macaques^16,25,26^. To our knowledge, however, an ability to mentally construct dual-structured symbols has not been reported in non-human animals except chimpanzees which used bigram and trigram Yerkish symbols^27–31^. Thus, evaluation of whether the macaques learn to construct dual-structured symbols or not would greatly advance our understanding of the evolutionary origin of mental spelling-like behavior in the primate lineage. To address this issue, we designed a unique symbolic bigram construction (SBC) task (Fig. 1 and Supplementary Fig. 1) for the macaques. Specifically, following acquisition of semantic and orthographic knowledge about the dual-structured bigrams by the macaques, the SBC task allowed evaluation of whether the animals were able to combine two elements of a bigram representing a visually-presented cue object while the bigram symbol itself was hidden. The hidden condition is critical because otherwise evaluation of whether the animal can mentally integrate semantic and orthographic knowledge should be impossible.

## Results

### Learning of dual-structured symbols

**Figure 2:**
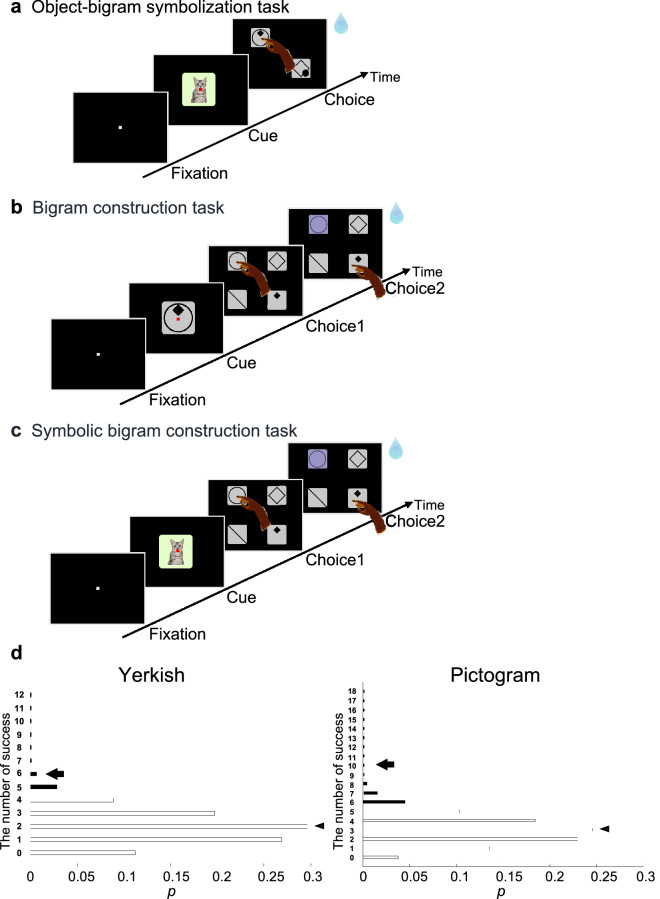
Symbolic construction of a meaningful bigram from meaningless elements in monkeys. (**a**) An object-bigram symbolization task (OBS task). A cue object and two bigrams are subsequently presented on the touch display. The monkeys are required to choose a bigram representing the cue object. (**b**) Bigram construction task (BC task). The monkeys must sequentially choose two elements that constitute the bigram presented as a cue. (**c**) Symbolic bigram construction task (SBC task). The monkeys are required to choose serially the two elements constituting the correct bigram that is not actually presented but is symbolically associated with the cue object. (**d**) The results of the SBC probe tests with the Yerkish (left) and pictogram (right) symbols, plotted on binomial distributions. Arrows indicate the observed number of successes out of 12 trials (left, for 6 stimuli in 2 animals) and 18 trials (right, for 6 stimuli in 3 animals), respectively. Bars represent the discrete probability distributions of the number of successes out of all probe trials. Arrowheads indicate the expected number of successes according to the binomial distribution, provided that the expected success rate is 0.17. Filled bars indicate the significance level of *p* < 0.050 with the one-tailed exact binomial test.

Macaque monkeys learned to symbolize six different objects with six bigrams, respectively, in the OBS task (Fig. 2a), with a success rate above the chance level of 0.50 for both Yerkish (monkey D, 0.91 ± 0.05; monkey S, 0.89 ± 0.03) and pictogram (monkey A, 0.91 ± 0.04; monkey D, 0.90 ± 0.05, monkey S, 0.92 ± 0.04) symbols (Supplementary Fig. 2).

Subsequently, in the BC task (Fig. 2b), monkeys learned to choose sequentially two elements of a visually presented bigram among four choices in any order. Training of the BC task was conducted with the identical symbols as those used in the OBS task until the performance exceeded the chance level of 0.17 for both Yerkish (monkey D, 0.77 ± 0.10; monkey S, 0.84 ± 0.05) and pictogram (monkey A, 0.81 ± 0.09; monkey D, 0.80 ± 0.09) symbols (Supplementary Fig. 2). However, monkey S was initially trained to learn the rule of the BC task with stimuli different from those used in the OBS task (bigrams made of geometric shapes) until the performance reached 0.87 ± 0.16 (Supplementary Fig. 2).

### Symbolic bigram construction in monkeys

From the initial probe trials of the SBC task (Fig. 2c) following training in the OBS and BC tasks, the monkeys could touch serially the two correct elements constituting the bigram that was not actually seen but symbolically associated with the cue object. As the chance level of success rate in the SBC task was 0.17, the expected number of successes out of 12 initial probe trials in the Yerkish symbol condition (for each of the 6 stimuli in 2 monkeys) was 2. The actual number of successes was 6 out 12 initial probe trials in the Yerkish symbol condition, the probability of which was significantly higher than expected by chance (one-tailed exact binomial test, *p* = 0.008, Fig. 2d, left). In the pictogram condition, the expected number of successes out of 18 initial probe trials (for each of the 6 stimuli in 3 monkeys) was 3. The actual number of successes was 10 out of 18 initial probe trials, the probability of which was also significantly higher than predicted by chance (*p* < 0.001, Fig. 2d, right). The number of successes out of 6 initial probe trials in individual animals was marginally significant (*p* = 0.063) in 4 conditions, and significant (*p* = 0.009) in one condition (Supplementary Fig. 3). The results indicated that once the monkeys acquired symbolic associations between the objects and the bigrams and the combination of elements in the bigrams were, they could mentally construct the bigram associated with the object even when the bigram itself was not presented as a clue.

### Development of spontaneous choice order bias

**Figure 3:**
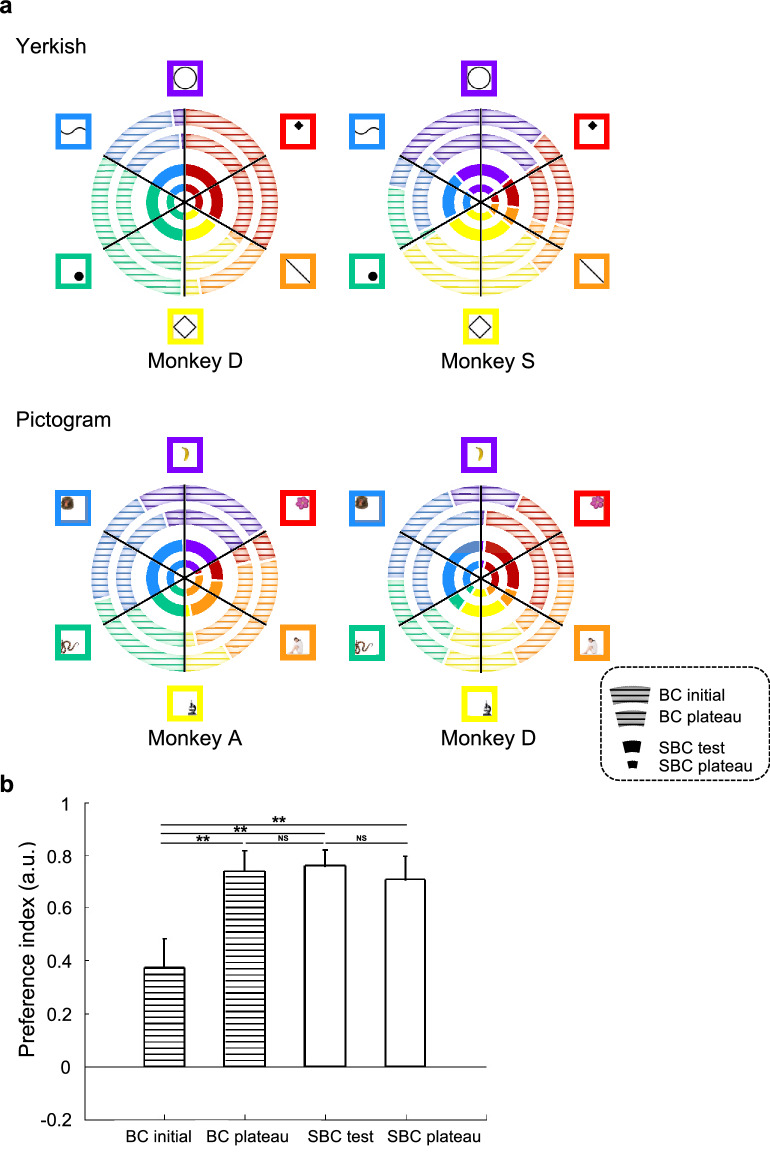
Development of choice order bias in individual monkeys. (**a**) Color charts indicating the preferred order whereby one of the paired elements constituting the Yerkish (upper) and pictogram (lower) bigram symbols is chosen first by individual monkeys. The striped outermost and the second outer circles indicate the choice order preference in the initial and plateau phases, respectively, of the BC task. The second inner and the innermost circles indicate the preference in the probe test and plateau phases, respectively, of the SBC task. Each of the six sectors is colored based on the probability that one of the paired elements is touched first by the monkeys (inset). (**b**) Preference index indicating the strength of the touch order bias (see “Methods”) in the initial and plateau phases of the BC task (striped), and the test and plateau phase of the SBC task (unstriped), respectively. The preference index for each task phase from all monkeys were pooled together and compared across task phases—BC initial vs. BC plateau, BC plateau vs. SBC test, SBC test vs. SBC plateau, BC initial vs. SBC test, and BC initial vs. SBC plateau, using a paired *t-*test with Bonferroni correction. Bars indicate standard errors of the mean (SEM) across 24 choice pairs for each monkey. **p < 0.01; *NS,* not significant.

Typically, when the bigram was a composite of a large white circle and a small black diamond, the probabilities of the circle and the diamond to be chosen first by monkey S were 0.70 and 0.30, respectively (Fig. 3a, upper right, outermost circle), which was not significantly different from the even distribution (χ^2^(30) = 4.80, *p* = 0.683, evaluated with one-tailed exact Chi-square test for goodness of fit with Bonferroni’s correction). Later in the plateau phase of the BC task learning, the order preference became prominent (Fig. 3a, upper right, second outer circle): the circle-first probability was 0.93 whereas the diamond-first probability was 0.07, and the order bias reached significance (χ^2^(30) = 22.53, *p* < 0.001). At the initial stage of the BC task, significant choice order bias was observed from the beginning for only 10 pairs (Fig. 3a, outermost circles). The number of pairs with a significant choice order bias increased through learning of the BC task. At the plateau phase of the BC task (Fig. 3a, second outer circles), 19 out of the 24 pairs exhibited significant choice order bias. Furthermore, analyses of the total trial numbers with preferred and non-preferred choice order for all the element pairs by one-tailed exact Chi-square test for independence (Supplementary Fig. 4) show that the choice order bias in the plateau phase of the BC task was stronger than in the initial phase (χ^2^(1, 720) = 70.00, *p* < 0.001; Fig. 3a, outermost *vs*. second outer circles). These results indicate that through the BC learning, the monkeys spontaneously developed and/or consolidated their own order preference in choosing the paired elements of the bigrams.

### Generalization of the acquired choice order bias across tasks

In the initial and plateau phases of the SBC task, 19/24 (Fig. 3a, second inner circles) and 18/24 (innermost circles) pairs, respectively, had significant choice order bias with one-tailed exact Chi-square test for goodness of fit, corrected with the aforementioned criterion. Notably, the preferred order was not significantly different between the plateau phase of the BC task and the initial phase of the SBC task (χ^2^(1, 720) = 0.41, *p* = 2.619; Fig. 3a, second outer *vs*. second inner circles and Supplementary Fig. 4).

The robustness of the monkeys’ choice order bias was quantified using a preference index (see “Methods”). Figure 3b shows that the preference index was significantly higher in the plateau phase (0.74 ± 0.08) than in the initial phase (0.37 ± 0.11) of the BC task (*d.f.* = 23, *t* = − 3.89, *p* = 0.004, two-tailed *t*-test, Bonferroni corrected for the number of *t*-tests). Importantly, the preference index in the initial phase of the SBC task (0.76 ± 0.06) was not significantly different from that in the plateau phase of the BC task (*d.f.* = 23, *t* = − 0.51, *p* = 3.065, Fig. 3b), but was significantly higher than that in the initial phase of the BC task (*d.f.* = 23, *t* = − 3.89, *p* = 0.004, Fig. 3b). The preference index of individual monkeys also exhibited the same tendency as the overall data (Supplementary Fig. 5). For all the monkeys/conditions, preference index was lower in the initial phase of the BC task compared to the other task phases and no significant difference was found between the BC plateau phase and SBC test phase. Thus, the choice-order bias consolidated during the BC task was maintained when monkeys began the SBC task, indicating that the monkeys might have imagined and mentally constructed the invisible bigram associated with the cue object during the SBC task.

## Discussion

In the present study we have shown use of dual-structured symbols by Japanese macaque monkeys (*Macaca fuscata*) resembling several fundamental features of human spelling. First, the macaques learned to symbolize artificial and natural objects including animals with arbitrarily assigned bigrams. Second, they learned to construct bigrams by choosing arbitrary combinations of two elements with a self-generated order. These results indicate that the macaques might acquire graphotactic and semantic knowledge of the bigrams, presumably by statistical learning. Third, the animals were able to construct the bigram that was invisible but symbolically specified by a cue object, with the same choice order bias as consolidated through the bigram construction learning. These results supported the idea that the monkeys should have recalled the visual image of bigram symbolically associated with the cue object and constructed the imagined bigram by mentally combining their elements. This implies that the macaques have the ability to mentally construct dual-structured symbols by integrating semantic and orthographic knowledge of the bigram. This ability of non-hominid animals might be in parallel with pre-linguistic spelling behaviors in human infants, which should be fundamental to linguistic development^7–12^. Therefore, our findings would be the significant bridge between comparative and developmental psychologies, shedding light on the evolution of human speech.

Two findings indicate that the macaques had acquired knowledge of the dual-structure of our visual symbol systems depending on statistical learning. First, in the OBS task, the animals should learn, by trial and error, arbitrary association between objects and symbols determined not by visual resemblance, but by temporal coincidence within the same trials. Second, the correspondence between the six bigrams and the six elements was such that each bigram was made of two discrete elements, whereas each element was shared by two different bigrams. We found that the performance of the SBC tasks was affected by the number of distractor elements that can be paired with one of the correct elements on other trials (Supplementary Fig. 6). This indicates that the macaques would have retrieved the two correct elements of the invisible bigram representing the cue object, but erroneously chosen one of the distractor elements depending on the probability of its occurrence with one of the correct elements in other trials. Therefore we speculate that the animals might have learned the arbitrary ties between the correct paired elements of the bigram depending on the statistical coincidence of the elements.

In both Yerkish and pictogram bigrams, different combinations of elements constituted meaningful symbols representing different objects. Yerkish symbols are similar to alphabetical words in that individual elements carry no semantic values by themselves. In contrast, individual pictograms originally symbolized artificial and natural objects including animals, as in Japanese *kana* letters. However, when paired with another pictogram as a bigram, the original meaning of the pictogram must be ignored. Therefore, compared to Yerkish, the use of dual-structured bi-pictograms requires an additional process to defy the original meaning of their elements. Despite this difficulty, the macaque monkeys exhibited a significantly above-chance performance in learning symbolic construction of both bi-pictograms and Yerkish bigrams. Thus, macaques can use symbols comprising pictograms that originally conveyed different meanings in isolation. A field study of wild putty-nosed monkeys (*Cercopithecus nictitans*) showed their ability to use a composite vocal sign signifying a relevant behavior in the wild environment by combining two elementary calls that had different meanings in isolation^32^. The macaques’ ability to acquire dual-structured bi-pictogram symbols in laboratory experiments might not be directly linked to the combination of vocal signs into a different sign in the wild. However, it is possible that macaques and putty-nosed monkeys might have evolved a proto-linguistic ability, in either visual or vocal form, to combine meaningful signs into a composite symbol with a different meaning defying the original meaning, from which humans would have evolved spoken and written language.

Provided that macaques can match arbitrary stimulus-stimulus associations^14,15^, there is a possibility that in the SBC task, each object might be newly associated with two elements through a complicated form of fixed conditional learning^24,33,34^, independent of the already acquired knowledge about bigrams. However, there are two reasons why the monkeys’ performance cannot be ascribed to rigid conditionality alone. First, the initial trial performance in the SBC probe test was significantly better than expected by chance. Significant learning generalization to the probe cues that the monkeys had never seen before excludes a possibility that they might have relearned individual associations between an object and two elements as new conditional rules in the SBC task. The result rather suggests that, in each trial of the SBC task, the animals should have retrieved the image of the hidden bigram specified by the cue object based on prior knowledge acquired through associative learning in the OBS task. Second, the spontaneous choice order bias acquired through BC learning was immediately transferred to the initial phase of the SBC task. The results exclude a possibility that the animals might have newly learned combinations of particular two elements by rigid conditionality in the SBC task, but rather suggest that knowledge about combinations of two elements constituting bigrams was immediately diverted in the SBC task. Taken together, these findings provide evidence suggesting that learning of the SBC task should not be ascribed to rigid conditionality. We conclude that, in the SBC task, the monkey would have integrated the semantic and orthographic knowledge of the hidden bigram associated with the cue object in each trial and constructed the recalled visual images of bigram by combining appropriate elements.

Among the design features of human language proposed by Hockett^3^, previous studies reported evidence for specialization, semanticity, arbitrariness, and discreteness^3^ in iconic symbol use by apes and macaques^14,15,35–37^. Primates can even recall hidden images from memory^14,35,37^, indicating a faint trace of displacement ability that signifies temporary and spatially remote referents^3^. Our findings are not only consistent with these results in that the macaques recalled hidden memories, but further provide evidence for use of symbols with duality of patterning^27–30^, at least in its simplest form, in non-hominid animals. This ability has been traditionally considered to be uniquely human. Thus, although the use of the non-verbal symbols by the macaques is dissimilar to spelling of human written language and lacks sound-to-letter interchangeability^3^, our findings have the potential to alter the way we think about the emergence of symbolization ability in primates.

The current findings in the macaques are consistent with previous researches reporting the ability to combine elements into a symbol in putty-nose monkeys^32^ and chimpanzees^27–31^, although the testing approaches were more or less different. Based on these findings, we propose that some biological adaptations in the general cognitive faculty would have occurred prior to the emergence of language. Specifically, common ancestors of human and Old World monkeys somewhere along the primate lineage might have acquired pre-linguistic adaptations for the modality-independent ability to combine meaningless elements into symbols. Such an adaptation would have been beneficial to increase the productivity, a design feature of human language which enables to generate virtually infinite number of messages by combining limited number of letters^1,3^. To the best of our knowledge, however, there has been no evidence for unlimited symbol production by non-human animals. The use of at most twelve or six dual-structured bigrams from limited combinations of elements cannot be considered on par with creative productivity of an infinite number of linguistic signs by combination of alphabets in human language. It also remains controversial whether non-human animal communication systems involve another design feature of human language called traditional transmission—across-generation transmission of linguistic traditions, not by genetically determined factors but exclusively by education and cultural learning^13,36,38–41^. It is difficult to examine whether and how the ability to use the dual-structured symbols by an adult monkey could be transmitted to younger generations under the laboratory conditions. Behavioral and neurophysiological evidence indicates that sequential hand motor actions associated with tool use have considerably evolved in non-human primates^42,43^, underpinned by homological neural networks with those of humans. Possibly, mental manipulation of symbols and objects would comprise the cognitive requirements for language development. Alternatively, the apparently comparable mental construction in humans, apes, and macaques might have been the results of convergent evolution, not based on homologous neural mechanisms. In other words, use of dual-structured symbols by the macaques might be underpinned by different neural networks from those underlying spelling in humans. To distinguish between these possibilities, it is critical to introduce neuroscientific technologies such as electrophysiology^35,44^ and chemogenetics^45^ into the macaque paradigms. Electrophysiology clarifies the correlation between cognitive behaviors and neural activity in high temporal resolutions in both macaques^35,44^ and humans^45^. Chemogenetics, which enables reversible, cell type-specific and pathway-specific modulation of neural activity via genetically engineered receptors, is one of most promising methods in assessing causality of neural activity to cognitive beheaviors^46^. The present study opens up the door for exploring the neural origin of spelling in the primate lineage.

## Methods

### Subjects

One female and two male Japanese monkeys (*Macaca fuscata*) weighing 5.1–9.8 kg, age 7–10 years old were used in the experiments. The animals were supplied by the National Bioresource Project “Japanese Monkeys.” Animals had been bred in a troop in the primate research institution from birth to weaning, which was similar to the wild environment. After 2–4 years of ages, they were moved to the laboratory environment. All animals were housed in standard primate cages under identical temperature and humidity conditions with a 14/10-h light/dark cycle and given primate food supplemented with fruits and vegetables. Animal housing was complied with the National Institute of Health Guide for the Care and Use of Laboratory Animals. All experimental protocols were approved by the Institutional Animal Care and Use Committee and the President of Niigata University, and were in compliance with the guidelines of National Institute of Health (NIH), Animal Research: Reporting of In Vivo Experiment (ARRIVE), and Japanese Ministry of Education, Culture, Sports, Science and Technology (MEXT).

### Dual structured visual symbol systems

We designed two sets of artificial visual symbol system with dual structure by modeling the dual structure of a written language (Fig. 1 and Supplementary Fig. 1). Each visual symbol system included six visual objects as referents, six bigram symbols, and six elements. The correspondence between the six bigrams and the six elements was such that each bigram was made of two discrete elements, whereas each element was shared by two different bigrams (Fig. 1c and Supplementary Fig. 1).

#### Visual objects

We selected two sets of six natural object images that the monkeys had never seen before as the referents of the Yerkish symbols (i.e., cat, bag, coffee, clock, airplane, maple) and the pictogram symbols (i.e., rat, avenue, planet, vehicle, guitar, and Japanese-kanji-character meaning “love”), respectively. To ensure that the monkeys could clearly distinguish all of the objects, we specifically selected six objects from six categories that were reported to be sufficiently apart from one another in hierarchical clustering analyses of perceptual and neural distances in previous macaque studies^47,48^. The object images were selected from a commercially available royalty-free picture library (Sozaijiten PhotoBible 20000, imagenavi, Hokkaido, Japan).

#### Bigram symbols

We used two systems: (1) six bigram Yerkish symbols^27,29–31^, each of which consisted of a combination of two meaningless elements of geometric shapes (Fig. 1c). and (2) six bi-pictogram symbols, each of which consisted of a combination of two elementary pictograms (Supplementary Fig. 1). Pictograms were selected on the same basis as visual objects. Pictograms originally symbolize artificial and natural objects including animals. Although each of the paired elements of a bi-pictogram symbol might have conveyed a different meaning in isolation, this original meaning must be ignored in the present experiments.

### Behavioral procedures

At the beginning of the experiments, the animals were habituated to sit voluntarily on the primate chair without any head or arm restraints (Nakazawa Co., Ltd, Tokyo, Japan; O'hara & Co., Ltd, Tokyo, Japan). Monkeys were trained in the object-bigram symbolization task (OBS) task, the bigram construction (BC) task until a criterion. The criterion was defined as *d'* > 1 in 50 consecutive trials. The *d'* value for a given trial was calculated from hit, miss, false alarm, and correct rejection ratios for each pair^49^ during a running time window of 100 trials. After monkeys’ performance reached the criterion, they underwent overtraining. In OBS and BC task, we selected 1000 trials from overtraining period to evaluate the monkeys’ performance for each monkey. Finally, the symbolic bigram construction (SBC) task was performed as a probe test.

In the OBS task, the monkeys underwent training to memorize associations between the objects and six bigram symbols as described previously^14,37^ (Fig. 2a). Each trial of the task started when the monkey pulled a lever following the presentation of a white square on a CRT display (Dv15a3, NEC, Tokyo, Japan) equipped with a touch screen (ELO touch systems, Tokyo, Japan). Subsequently, a cue object was presented on a green panel (10° × 10° in visual angle) in the center of the display for 1–2 s. Following a 0–1 s delay, two choice stimuli, a correct bigram signifying the cue and a distractor, appeared on gray panels (8° × 8°) in two of the four peripheral positions in the display. The animal was required to touch the correct choice bigram to obtain a drop of liquid reward (fruit juice). A total of 800–1200 trials were conducted in a typical daily training session. The timing, synchronization, and data storage of the behavioral experiments were controlled by a custom-made software running on a Windows-based PC system (Presentation, Neurobehavioral System, Albany, USA). After reaching a criterion (see below) and overtraining, the monkeys were trained in the BC task (Fig. 2b). In this task, one of the six bigrams was presented as a visual cue and after a 0–1 s delay four elements were presented as choices on the display. The animals were required to choose sequentially a correct pair of elements constituting the bigram. In the pictogram symbol condition, monkey S was trained in the BC task with different symbols as those used in the OBS and SBC tasks. Thus, the pictogram condition in monkey S was excluded from the analyses of choice order bias or preference index.

Finally, a probe test in the SBC task was conducted. On each trial of the SBC task (Fig. 2c), one of the six objects was presented as a cue; and after a 0–1 s delay four elements were presented as choices on the display. Among the four choice elements, the monkeys were required to touch sequentially the correct two elements constituting the bigram symbol that was not actually seen but specified by the cue object. If the first touch was correct, the color of the chosen element turned dark, informing the animal that this element was no longer the second choice target. The monkey obtained a reward when the second element was also correctly touched. The SBC task required combinatorial responses, specifically that two out of four alternatives; hence the chance level was 0.17. The type and amount of reward during the training remained constant across stimuli and the number of exposures to each stimulus was counterbalanced across trials. Thus, the reward value for individual stimuli was not particularly biased in our paradigm. The position of the choices and distractors are also counterbalanced across trials. We examined whether the initial trial performance for each object in the probe test was significantly different from the chance level predicted by the binomial distribution. In the first six trials, each combination of an object and two correct choices was presented only once, excluding a possibility that a complex form of conditional learning might have occurred during the probe test. After the probe test, six objects were presented in a random order for overtraining.

The same training procedures and criteria were used for the behavioral experiments with Yerkish and pictogram symbol systems. Monkey A was used in a pilot behavioral and electrophysiology experiment with pictogram symbol system alone, after completion of which was perfused transcardially for histological examination. Thus, there was no data from monkey A for Yerkish symbols.

### Data analysis

#### The probe test of the symbolic bigram construction task

We assessed the monkeys’ performance in the probe trials of the SBC task based on the number of successes out of the first six trials, in each of which the monkey was tested with one of the 6 probe cues (Fig. 2d and Supplementary Fig. 3). Data from all monkeys were averaged for both Yerkish probability (monkey D and S) and pictogram (monkey A, D and S) symbol systems. Since the animals were required to choose two elements among four choice elements, the expected success rate by chance was 0.17 in each trial of the SBC task. We used the binomial test to evaluate the statistical significance of deviations of the observed number of successes out of 12 or 18 probe trials from the theoretically predicted binomial distribution.

#### Voluntary choice-order analyses

The data from BC and SBC task were used for voluntary choice-order analyses. Data from two monkeys (D and S) with six Yerkish bigrams and data from two monkeys (A and D) with six bi-pictograms—in total 24 (6 × 2 × 2) element pairs—were pooled for the analysis. A Chi-square test for goodness of fit was performed to evaluate whether the probability of a particular element to be chosen first by a monkey was significantly different from the chance level of 0.50 (Fig. 3a).

In addition, the Chi-square test for independence was performed to examine whether the choice bias of each task phase has significantly difference (Fig. 3a and Supplementary Fig. 4). All monkeys’ data were pooled together to calculate the number of trials with preferred and non-preferred order in each task phase for comparison across task phases.

#### Preference index

The preference index was used to quantify the choice order bias for each task phase (Fig. 3b and Supplementary Fig. 5). The preference index was calculated using the following equation: preference index = (N1 − N2)/(N1 + N2), where N1 and N2 indicate the number of trials with the preferred and non-preferred choice orders, respectively. This index indicates the degree to which the touch order for a given element pair is biased. The preference index for each task phase from all monkeys were pooled and compared across task phases.

All *p*-values were corrected using the Bonferroni correction for Chi-square tests and *t*-test. All analyses were performed using a custom-coded program with MATLAB (MathWorks, MA, USA) with the Statistics Toolbox.

## Supplementary Information

Supplementary Figures.

## Data Availability

The data that support the findings of this study are available from the corresponding authors upon reasonable request.
