# Supplementary material for: Mental construction of object symbols from meaningless elements by Japanese macaques (Macaca fuscata)
Source: Sci Rep. 2022 Mar 4;12:3566. doi: 10.1038/s41598-022-07563-z (PMC8897398; doi:10.1038/s41598-022-07563-z)
Supplement: Supplementary file 1 — Supplementary Figures. [file 41598_2022_7563_MOESM1_ESM.docx]

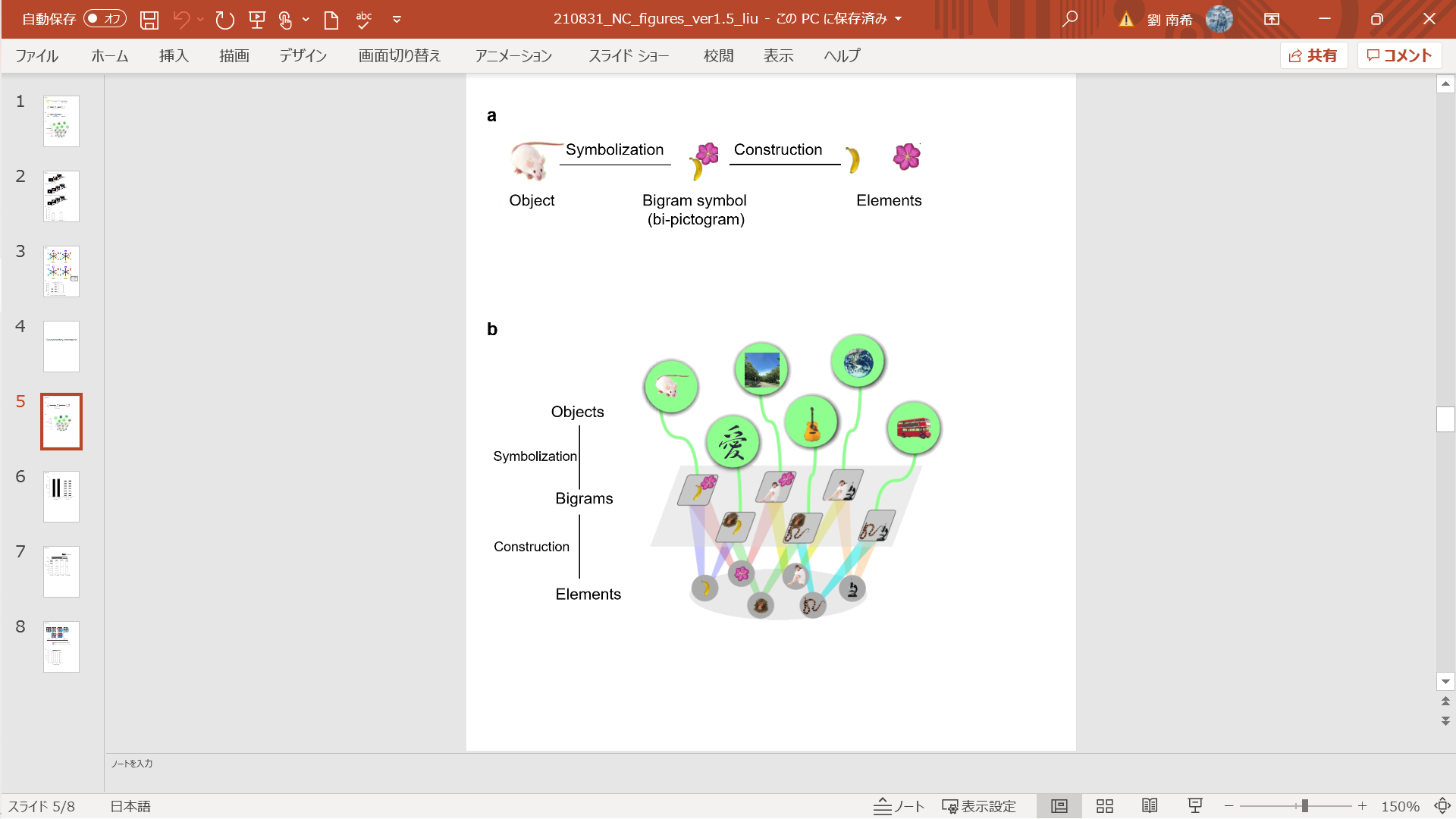


**Supplementary Figure 1. A dual-structured visual symbol system with pictograms.**

**a** A bi-pictogram serves as a meaningful unit representing an object and is a combination of two elementary pictograms the original meaning of which must be ignored in the present experimental paradigm.

**b** A dual-structured visual symbol system with pictograms. Six objects (upper) are represented by six arbitrary bi-pictograms (middle), which comprise different combinations of elementary pictograms (lower).


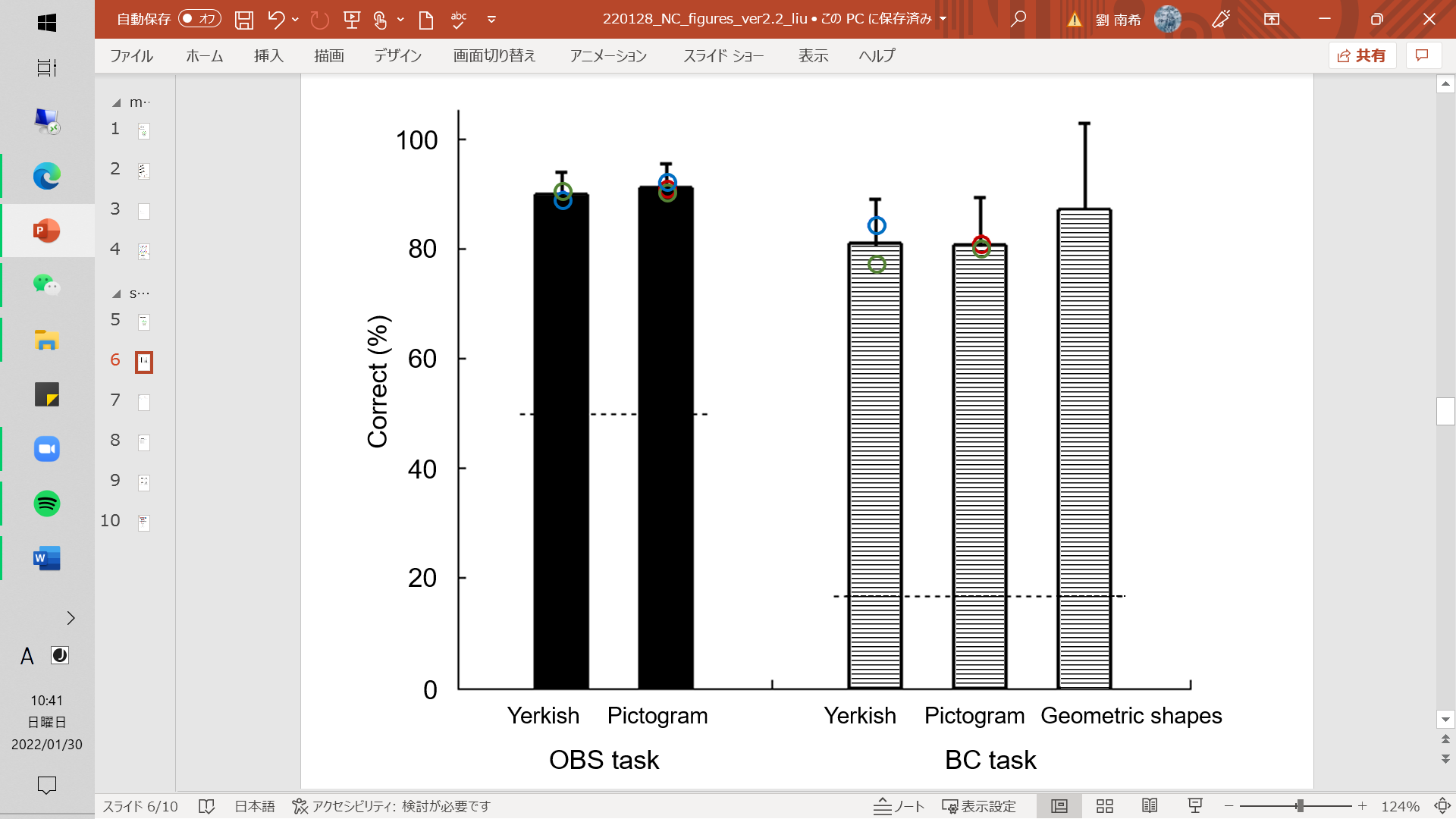


**Supplementary Figure 2. OBS task and BC task performance with Yerkish and pictogram symbol systems.**

Bars represent performance of the OBS (black) and BC (striped) tasks, respectively, averaged across monkeys. Circle marks indicate the performance of monkey A (red), D (green) and S (blue), respectively. Performance was measured during the overtraining phase after the monkeys reached a criterion in 4–16 days (Yerkish, 4–15 days; pictogram, 7–16 days) and 10–31 days (Yerkish, 10–26 days; pictogram, 11–31 days; geometric shapes, 1-3 days) of training, respectively in the OBS and BC tasks. Dashed lines indicate the chance level of the OBS (0.50) and BC (0.17) tasks. Error bars indicate standard deviations across monkeys and objects.


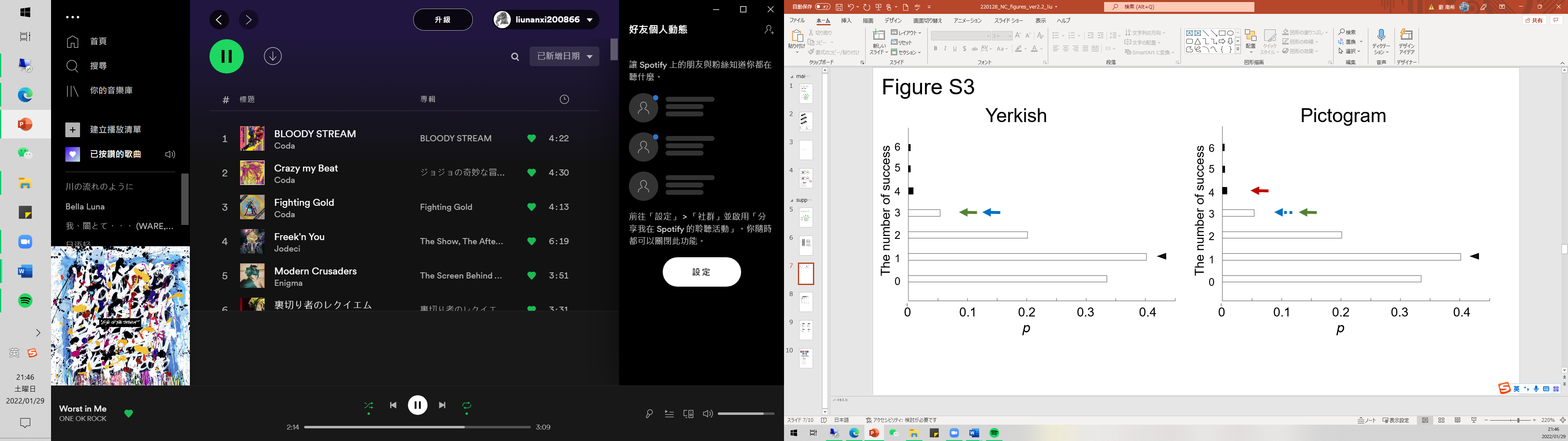


**Supplementary Figure 3. The number of successes in the SBC probe tests in individual monkeys, plotted on binomial distributions.**

The probe test of the SBC task was conducted for each of the 6 Yerkish (left) or pictogram (right) symbols after training in the BC task with the identical symbols, except monkey S’s pictogram condition (blue dotted arrow) which followed training of the BC task rule with different stimuli (geometric shapes). In the Yerkish condition, the number of successes out of 6 probe trials was 3 for both monkeys D (green arrow) and S (blue arrow). Provided that the chance level of success rate in the SBC task was 0.17, the probability of 3 or more successes out of 6 trials was marginally significant (one-tailed exact binomial test, *p* = 0.063) with the binomial test. In the pictogram condition, 4 out of 6 trials were successful for monkey A (red arrow), the probability of which was significantly higher than expected by the chance (*p* = 0.009). Probability of successful trials in monkeys D (3/6) and S (3/6) were marginally significant (*p* = 0.063). Arrowheads indicate the expected number of successes. Filled bar indicates the *p* < 0.050.


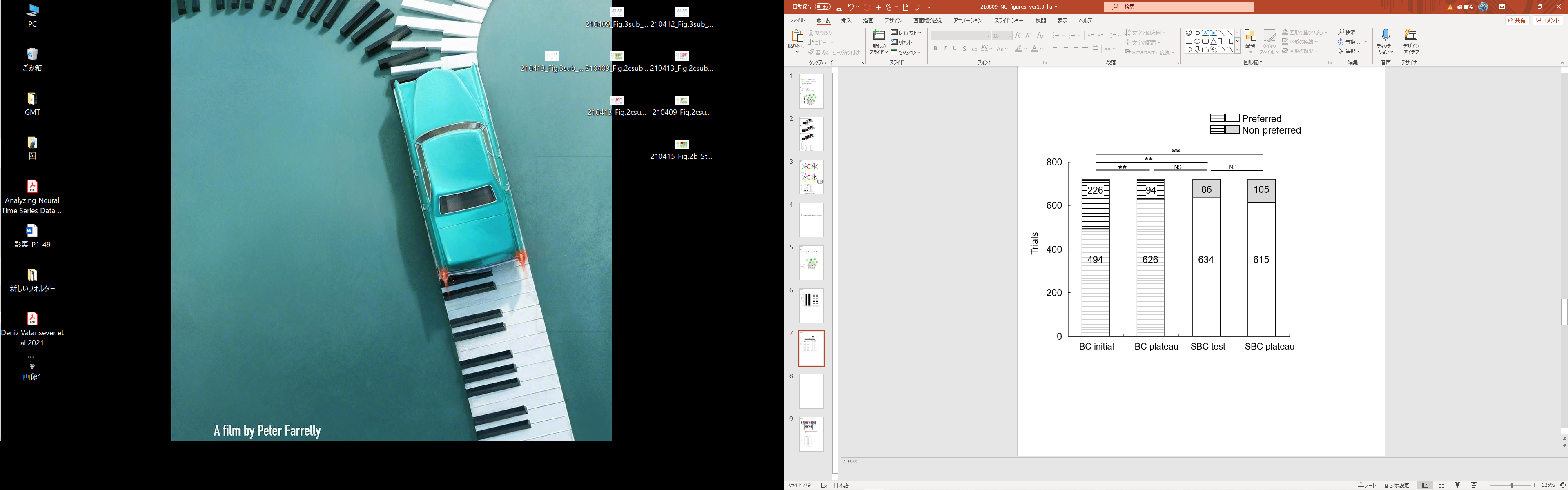


**Supplementary Figure 4. Number of trials with preferred and non-preferred choice order during different task stages.**

Trial distribution of the preferred (white) and non-preferred (gray) orders in the initial and plateau phases of the BC task (striped) and the probe test and plateau phases of the SBC task (unstriped). The preferred and non-preferred orders were defined based on the choice order in the test phase of the SBC task (see Methods). All monkeys’ data were pooled. One-tailed exact chi*-*square test for independence was used for comparison across task phases— BC initial vs. BC plateau, BC plateau vs. SBC test, SBC test vs. SBC plateau, BC initial vs. SBC test, and BC initial vs. SBC plateau. *p*-value was corrected using Bonferroni correction with the number of tests. ***p* < 0.010; NS, not significant.


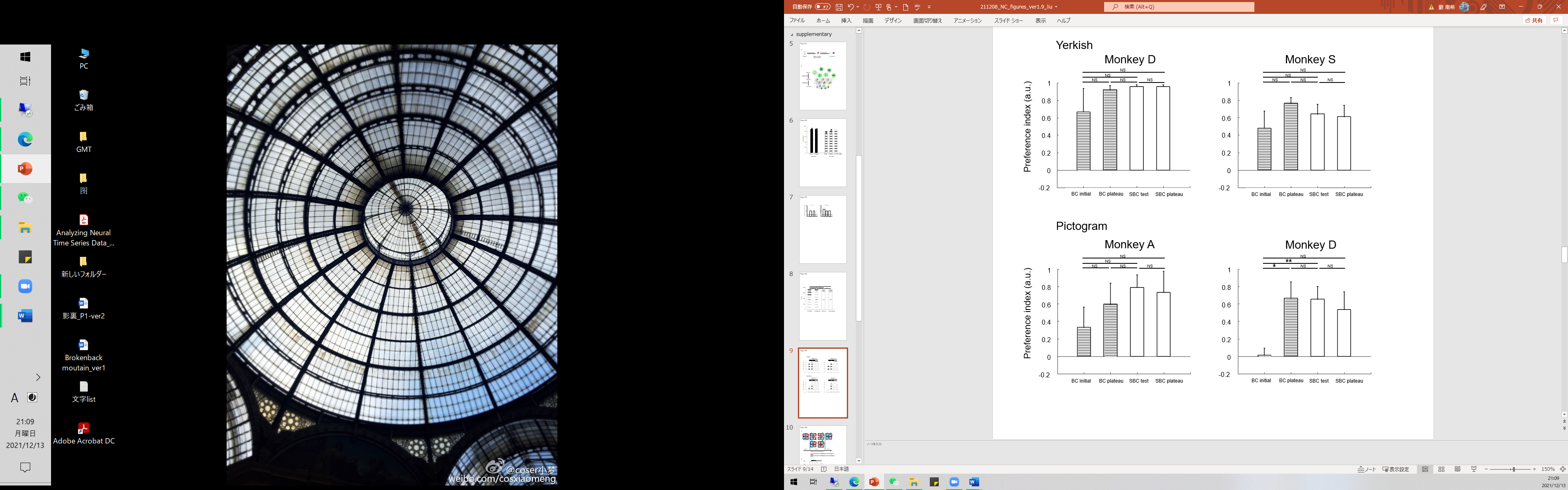


**Supplementary Figure 5. Preference index in individual monkeys.**

For all monkeys/conditions, the preference index was the lowest in the initial phase of the BC task, and there were no significant differences between the BC plateau and the SBC test phases. Formats are as in Figure 3, except those bars indicate standard errors of the mean (SEM) across 6 choice pairs, instead of 24 pairs, for each condition. **p* < 0.050, ***p* < 0.010 in two-tailed *t-*test with Bonferroni correction for the number of *t*-tests. NS, not significant.


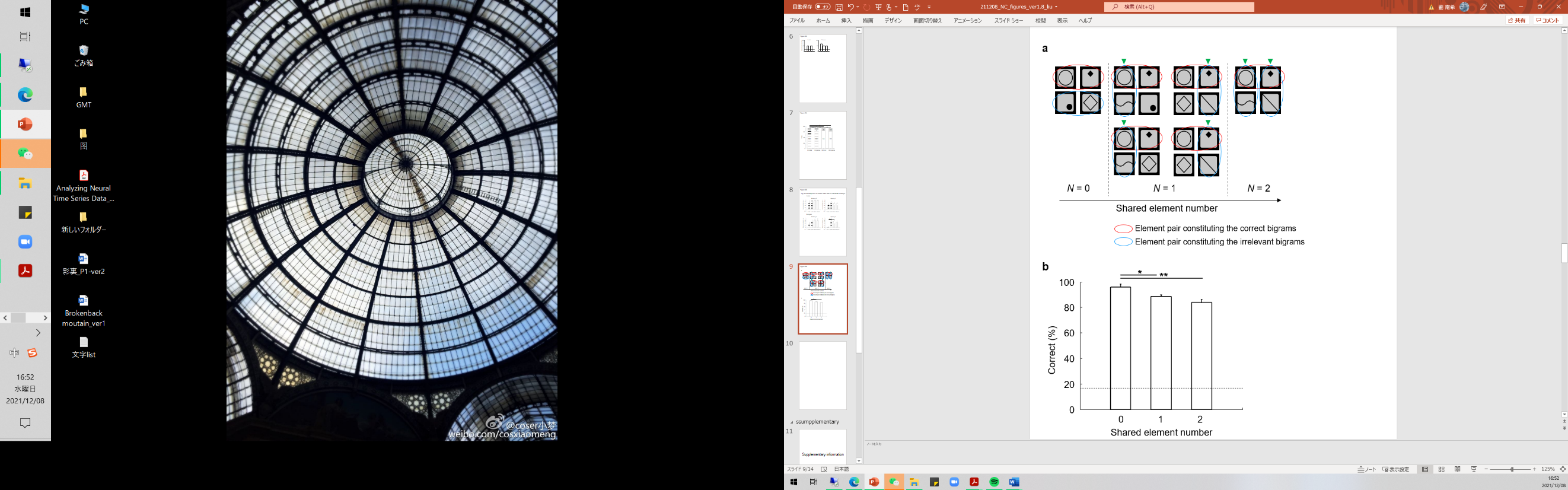


**Supplementary Figure 6. Dependency of the SBC performance on the number of elements shared by the correct and irrelevant bigrams during the choice period.**

**a** In the SBC task, the shared element number, *N*, was defined as the number of elements shared between the correct bigram for a particular trial (red dashed ellipse) and bigrams irrelevant for that trial (blue dashed ellipses) in the configuration of the four elements during the choice period. Green arrowheads indicate the shared element.

**b** Monkeys’ performance in the plateau phase of the SBC task sorted with the shared element number defined in **a**. The dashed line indicates the chance level 0.17. Bars indicate the SEM across 14 sessions. * *p* < 0.050, ** *p* < 0.010, one-way ANOVA and *post-hoc* tests with Holm-Bonferroni correction.
